# Performances of the WEPP and WaNuLCAS models on soil erosion simulation in a tropical hillslope, Thailand

**DOI:** 10.1371/journal.pone.0241689

**Published:** 2020-11-04

**Authors:** Wattanai Onsamrarn, Natthapol Chittamart, Saowanuch Tawornpruek

**Affiliations:** 1 Department of Soil Science, Faculty of Agriculture, Kasetsart University, Chatuchak, Bangkok, Thailand; 2 Center for Advanced Studies for Agriculture and Food, Institute for Advanced Studies, Kasetsart University, Bangkok, Thailand; Imperial College London, UNITED KINGDOM

## Abstract

Effective soil erosion prediction models and proper conservation practices are important tools to mitigate soil erosion in hillside agricultural areas. The Water Nutrient and Light Capture in Agroforestry Systems (WaNuLCAS) and Water Erosion Prediction Project (WEPP) models are capable tools in soil erosion simulation in the conventional and conservation cropping systems in hillslopes. We calibrated both the models in maize monocropping and simultaneously validated them in maize-chili intercropping with *Leucaena* hedgerow for nine rainfall events in 2010, with the aim to evaluate their performances in runoff and sediment prediction on a skeleton soil in a hillslope, Western Thailand. The results showed that the calibrated WaNuLCAS model poorly predicts runoff prediction in the validation. In contrast, the calibrated WEPP model had a better performance in runoff prediction in the validation. For sediment prediction, the calibrated WaNuLCAS model predicted sediment yield better than the calibrated WEPP model in the validation because the WEPP model shows more variability of the sediment yield in the calibration (5.84 kg m^–2^) than the WaNuLCAS (5.18 kg m^–2^). Thus, the WEPP model was more suitable for runoff prediction than sediment prediction in the monocropping system, whereas the WaNuLCAS model was better suited for sediment yield prediction than runoff prediction, especially in complex intercropping systems.

## Introduction

The recently adopted United Nations Sustainable Development Goals (SDGs) suggest that the sustainability of human societies relies on the appropriate use of natural resources, especially soils [1]. The soil system is a key that controls biological, hydrological, erosional, and geochemical earth cycles of the earth system [2, 3]. Soils play an important role in supplying water, nutrients, and a substrate to plants, and soil properties influence agroecosystems and natural health [4, 5] including human health [2]. However, soil erosion processes have been a major agricultural concern; agricultural land use is associated with the highest erosion rates and causes more sediment yield compared to that in other land uses. Key factors that contribute to soil erosion are soil disturbance, tillage, removal, lowering of the overall vegetation cover, diminishing organic matter, soil compaction, sealing, and the conservation practice in sloping terrains [6–10].

Moreover, soil erosion is a major environmental challenge in terms of sustainability and agricultural productive capacity of the land [11, 12]. It is estimated that approximately 10 million hectares of global cropland are lost to erosion annually [11, 13]. Further, the estimated world population may increase to 10 or 12 billion by 2100, mostly in developing countries. The rapid increase in the population has resulted in the conversion of natural ecosystems to agricultural land uses on large scale to cater to the growing food demands [14]. The declining soil productivity and fertility, which represent the most significant “on-site impacts” of soil erosion, are most common in the tropical and subtropical agro-ecosystems of Asia, Africa, and South America, where soil loss averages 30–40 t/ha/year [15]. Currently, in Thailand, farmers are encountering low prices of agricultural products, climate change, and seasonal disasters. Deforestation for farming cultivation resulted in approximately 34% of soil erosion of the agricultural area, especially in hillside areas, in Thailand. Therefore, good land management would play an essential role to preserve soil functions, and it is required to achieve the desired sustainability of the country.

Nowadays, land use and soil cover are considered the most important factors affecting the intensity and frequency of runoff and soil erosion [16]. With respect to three simultaneous erosion processes—detachment, transportation, and deposition—the most widely applied techniques of soil management combined with agronomic practices to reduce soil erosion (i.e., intercropping, agroforestry, cover crops, animal manure, contour farming, minimum tillage, terraces, windbreaks, and hedges) are reviving soil fertility and enhancing crop production [17]. Recent studies demonstrated that soil surface mulching with plant residues can avoid high runoff and erosion rates in eastern Spain [18] and Mediterranean rainfed agriculture land [19]. Further, contouring grass strips or hedgerows are effective soil conservation practices for reducing runoff and soil erosion on slope areas [20–22]. On the other hand, crop rotation between maize-cowpea incorporating *Leucaena* hedgerow has effectively reduced runoff and soil erosion by 64% and 85%, respectively, as compared to conventional tillage treatment in Vietnam, which demonstrates that applying hedgerow and grass barriers effectively controlled soil erosion in the tropical situation [17]. Moreover, a combination of maize cropping with minimum tillage and Jack bean relay cropping could increase crop yield over time, even without fertilization, and improve soil erosion in the third year in northern Thailand [22]. Thus, the contouring hedgerow and crop rotation can be attributed to soil erosion control by acting as a plant barrier to hamper surface runoff water, and the pruned plant residues mulching on soil surface retarded raindrop impact to the soil surface. However, the applicability of cropping systems and land management to control soil erosion and runoff is site-specific and dependent on climatic conditions (*i*.*e*., total rainfall and intense rainfall events) [23]. Nevertheless, field trials and domain tools to monitor the effect of conversion of forest to agriculture land, and introducing cropping systems on soil erosion are time-consuming, costly, and laborious. Thus, crop and soil models are tools required for examining and improving the efficiency and performance of soil and water management practices and for saving the soil management cost [24]. On the other hand, soil erosion models also accomplish both soil conservation practices and the scientific understanding of soil erosion processes.

In general, soil erosion models can be classified into empirical and physically based models [25]. Empirical models generate outputs that are linked to the inputs by some sort of a mathematical equation. Usually, the data derived from large regional databases that limit the applicability for some other regions. Whereas, physically-based models refer to an individual mechanism process in the component is produced and analyzed within the model. Therefore, it can perform a wider range of applications and provide better temporal and spatial assessments of erosion processes in various land-use scenarios [26–28].

The Water Erosion Prediction Project (WEPP) model is a physically-based continuous simulation model for predicting water erosion and sediment yield and deposition from rainfall, snowmelt, and irrigation as spatially and temporally distributed processes [29, 30]. WEPP has been tested and applied for simulating runoff and soil losses in various geographic locations in the United States [31–34], in Australia [35], in the United Kingdom [36], in a hilly area of Italy [37] and in the Emameh watershed in North-West of Tehran, Iran [38]. On the other hand, the Water, Nutrient and Light Capture in Agroforestry Systems (WaNuLCAS) model simulates dynamic processes in the spatial domain. It was developed to represent Tree-Soil-Crop interactions under a wide range of agroforestry systems where trees and crops overlap at the plot level. This was successfully applied to simulate the effects of soil conservation practices on soil loss and runoff, soil structure, and water infiltration in Northeast Thailand [39], to evaluate the impact of improved fallows on maize yield under various soil and environmental conditions of Kenya [40] and to assess limiting factors at the crop-soil-hedge interface reducing maize aboveground biomass in rows close to hedgerows in Western Thailand [41]. As the WaNuLCAS and WEPP models have the capability to simulate various cropping system patterns to evaluate land use and management in a single simulation, the models have not previously been tested on a small-plot scale (e.g., hillside agricultural area) under an erratic tropical rainfall region in Thailand. Therefore, both models were selected to be tested in this study to evaluate their potential to be used as a tool for soil erosion prediction in the hillslope soils of Thailand and other tropical regions. This paper also discussed the performances of both models in the prediction of runoff and soil loss under conventional and conservation practices on a stony soil in a tropical hillslope, western Thailand. Thus, the objectives of this study were: (i) to evaluate the performance of the WaNuLCAS and WEPP models in the simultaneous prediction of runoff and soil loss under different crop management techniques and (ii) to compare the performance of both models for runoff and sediment prediction under different crop management and farming practices.

## Materials and methods

### Experimental site description

The experimental site was implemented at the Demonstration Farm under Patronage of Her Majesty the Queen Sirikit in Bo-Wi village, Suan Phueng district, Ratchaburi province, Thailand (13°28′8.20″N Latitude, 99°16′0.20″E Longitude). All field activities and equipment installation were permitted by the Office of the Royal Development Projects Board. The demonstration farm was established as an open center for learning by doing, food production, study and development in agriculture for people in Suan Phueng district and surrounding areas. The experimental area did not involve endangered or protected species. The data logger (CR1000: Campbell Scientific Inc.) was installed in the experimental site for collecting meteorological data based on an hourly and daily basis. The mean annual precipitation during 2010 was 1,220 mm, where the highest rainfall volume in the experimental site was in the period of July to September. The mean air temperature was 27.5°C with a maximum temperature of 41°C in April and a minimum of 10°C in January. The uneven slope gradient ranges from 6–12%. The soil is shallow with 80 cm depth and contains many phyllitic and quartzitic rock fragments with an average value of 44.7–85.3% w/v and low organic matter content (4.1–8.0 g kg^–1^) within the soil profile. The soil in the experimental site was classified as a Loamy-skeletal, siliceous, isohyperthermic, Kanhaplic Haplustults according to USDA Soil Taxonomy. Soil texture within the soil profile is slightly to extremely gravelly sandy loam to clay loam. Soil pH (1:1 H_2_O) is acid to strongly acid (4.5–5.8). This field site has been previously described by Garré et al. [42] and Khetdan et al. [43].

### Soil and cropping system management

**Fig 1:**
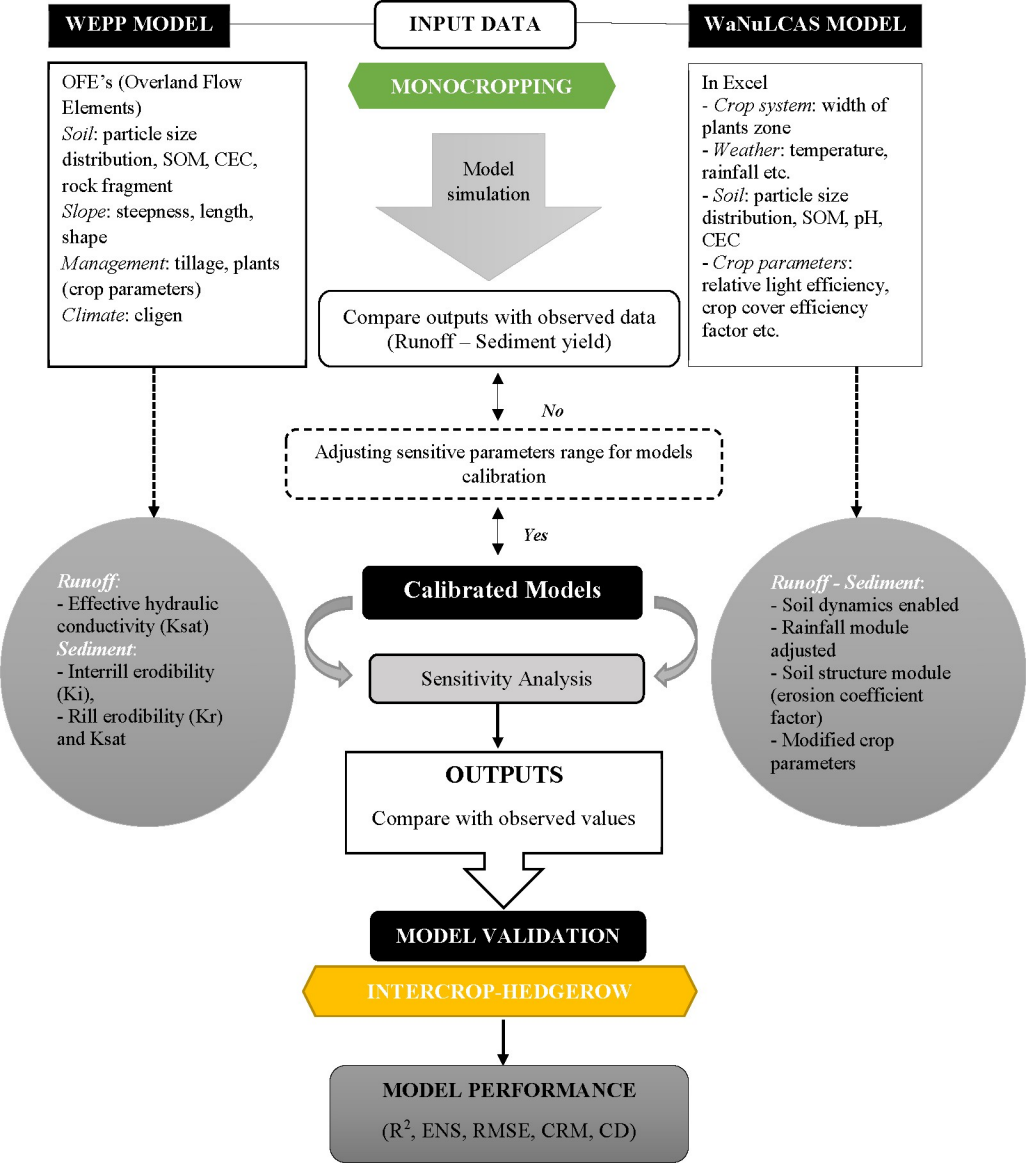
Protocol for calibration and validation of the WEPP and the WaNuLCAS.

The data of two treatments with three replications of conventional and conservation cropping systems practices on a hillslope were used for the calibration and validation of the WEPP (version 2101.8) and WaNuLCAS (version 4.0 running in the STELLA 8) models. The monocropping with conventional tillage (15–20 cm depth) (so-called “monocrop”) represents the conventional practice. The intercropping with hedgerow (so-called “intercrop-hedgerow”) represents the conservation practices, with which maize was intercropped with chili, minimum tillage (<10 cm depth), and interval strips of *Leucaena* hedgerow (Fig 1). The main crop of each cropping system was maize (*Zea mays*, CV Pacific 999), intercropped plant was high-value chili (*Capsicum frutescens* L. CV Superhot) and hedgerow was *Leucaena* (*Leucaena leucocephala*). Urea (46-0-0), triple superphosphate (0-46-0) and potassium chloride (0-0-60) were applied to maintain normal growth of maize. The plot size of each cropping system was 52 m^2^ (4 m wide × 13 m long) and was aligned along the slope gradient on the hillslope. Runoff and soil sediment were collected through the ditches installed at the end of plots and connecting to the water container. Runoff and sediment were collected after every single storm that produced runoff discharge and sediment for the whole years.

In the monocrop practice, maize was seeded by the end of June 2010 with a spacing of 0.25 m within maize rows and 0.75 m between maize rows. In the intercrop-hedgerow practice, the spacing between *Leucaena* hedgerow and maize rows was 0.25 m, the spacing between maize rows was 0.75 m, and the spacing between maize rows and chili was 1.0 m.

### Input data for model simulations

The required input data in the model simulation were climatic data from the data logger installed in the site, soil properties, plant growth, and management practices. In the WEPP model, four data files were required to represent overland flow elements (OFEs) for simulation including slope element which was measured from the experimental plots. Climatic data includes daily precipitation, maximum and minimum air temperature, solar radiation, wind velocity and direction, and dew point temperature. Analytical soil properties given in Table 1 were used for the soil module of the WEPP models including soil particle size distribution and rock fragments percentage, cation exchange capacity (CEC), and soil organic matter content (SOM). For interrill erodibility (K_i_), rill erodibility (K_r_), critical shear stress (ρa), and effective hydraulic conductivity (K_sat_) were able to calculate following the manual in the WEPP technical documentation or by default of the model calculation. In the management file, the calendar of activities including planting, tillage, and irrigation are in the database that was adjustable to fit with the simulated site. In addition, the crop parameters database in the management file was adjusted or selected based on default values from the database library.

**Table 1:** Soil parameters input in the WEPP model for simulation processes.

Soil horizon (cm)	Particle size distribution			Chemical properties		Rock fragment content
	Sand	Silt	Clay	SOM	CEC	
	(%)	(%)	(%)	(%)	(cmol_c_ kg^-1^)	(%/Vol.)
Soil parameters in monocropping system (monocrop)						
0–5	40.5	37.8	21.7	2.1	12.3	17.3
5–15	37.1	41.2	21.7	2.1	12.3	23.8
15–45	43.5	32.6	23.9	1.6	10.0	51.2
Soil parameters in intercropping with hedgerow system (intercrop-hedgerow)						
0–5	41.4	37.5	21.1	2.0	10.6	17.3
5–15	40.1	41.9	18.0	2.0	10.6	23.8
15–45	43.7	37.0	19.3	1.4	8.0	51.2

For the WaNuLCAS model, the input data in the Excel spreadsheet require weather data that consist of daily precipitation, soil temperature, and evaporation. Pedotransfer was calculated using a percentage of sand, silt, clay, organic carbon, and soil bulk density (Table 2). Crop schedule and crop parameters (Table 3) for each zone were in the crop management and crop library spreadsheet.

**Table 2:** Soil parameters used for the pedotransfer sheet in the WaNuLCAS model for simulation processes.

Soil horizon (cm)	Particle size distribution			Chemical properties			Rock fragment content	Effective hydraulic conductivity (K_sat_)
	Sand	Silt	Clay	SOM	CEC	Soil pH		
	(%)	(%)	(%)	(%)	(cmol_c_ kg^-1^)		(%/Vol.)	(Mg m^-3^)
Soil parameters in monocropping system (monocrop)								
0–5	40.5	37.8	21.7	2.1	12.3	5.7	17.3	Calculated by the models
5–15	37.1	41.2	21.7	2.1	12.3	5.9	23.8	
15–30	43.5	32.6	23.9	1.6	10.0	5.6	51.2	
30–45	43.5	32.6	23.9	1.6	10.0	5.6	51.2	
Soil parameters in intercropping with hedgerow system (intercrop-hedgerow)								
0–5	41.4	37.5	21.1	2.0	10.6	5.6	17.3	Calculated by the models
5–15	40.1	41.9	18.0	2.0	10.6	5.6	23.8	
15–30	43.7	37.0	19.3	1.4	8.0	5.7	51.2	
30–45	43.7	37.0	19.3	1.4	8.0	5.7	51.2	

**Table 3:** Crop parameters input in crop library of the WaNuLCAS model for the simulation processes.

Parameters (Maize)	Default	Calibrated
Length of generative stage	30	63
Length of vegetative stage	60	50
Earliest day to flower in a year	1	225
Latest day to flower in a year	365	253
Production of dry matter per day	0.014	0.1
Seed weight	0.004	0.1
Water requirement for dry matter production	300	30
The maximum proportion of crop biomass remobilized as the storage component	0.05	0.01
Extinction light coefficient	0.65	0.68
Maximum Leaf Area Index	5	6
Rainfall water stored at the leaf surface	1	0.01
Max. root length density in layer 1	5	2
Max. root length density in layer 2	3	1
Max. root length density in layer 3	0.3	0.5
Crop cover efficiency factor	0.3	0.1
Standard moisture content	0.15	0.5
Cq_RelLUE = Relative light use efficiency (dimensionless)	default	multiplied by 0.4
Cq_SLA = Specific Leaf Area (m^2^/kg)	default	multiplied by 0.7
Cq_LWR = Leaf Weight Ratio (dimensionless)	default	multiplied by 0.84

### Model performance assessment

The model performances of two model simulations were evaluated by comparing the simulated values against the observed data of the runoff and sediment yield collected during the growing season in the year 2010. The coefficient of determination (R^2^) was used to measure how close it is to a linear 1:1 relationship between the observed and simulated outputs. Several specific statistical equations [30, 44, 45] were also used for improving the assessment of the model performance.

Modeling efficiency (*E*_*NS*_) [44] was calculated to assess the goodness of fit. A value of 1.0 demonstrates an absolute one-to-one correspondence between the simulated and observed values. The range of *E*_*NS*_ values is from -∞ to 1, with 1 indicating a perfect fit. Values between 0.0 and 1.0 are commonly considered as acceptable for model performance and values <0.0 indicates that the mean observed value is a better predictor than the simulated value, which indicates unacceptable model performance [46]. For values of *E*_*NS*_ > 0.75, the model simulations are considered to be good and E_*NS*_ > 0.5 are considered to be acceptable [47, 48] and for values between 0.36 and 0.75, the model simulations are considered satisfactory [48]. However, a shortcoming of the Nash–Sutcliffe statistic is that it does not perform well in periods of low flow. If the daily measured flow approaches the average value, the denominator of the equation tends to zero and *E*_*NS*_ approaches negative infinity, with only minor prediction errors of the model. This statistic works well when the coefficient of variation for the data set is large [49].

$$E_{NS}=1-\frac{\sum_{i=1}^{n}{\left(Oi-Pi\right)}^{2}}{\sum_{i=1}^{n}{\left(Oi-\bar{O}\right)}^{2}}$$

The coefficient of determination (CD) is a measure of the proportion of the total variance of observed data explained by the simulated data; a value of one indicates an absolute prediction fit.

$$CD=\frac{\sum_{i=1}^{n}{\left(Oi-\bar{O}\right)}^{2}}{\sum_{i=1}^{n}{\left(Pi-Ō\right)}^{2}}$$

The root means square error (RMSE) is expressed in percentage to designate the average error of simulated results where the value of zero expresses the goodness of the agreement between observed and simulated data [50].

$${RMSE=[\sum_{i=1}^{n}{\left(Oi-Pi\right)}^{2}/n]}^{0.5}$$

The coefficient of residual mass (CRM) is a measure of the tendency of the model to overestimate or underestimate the measurements. A negative CRM shows a tendency to overestimate.

$$CRM=\frac{\sum_{i=1}^{n}Oi-\sum_{i=1}^{n}Pi}{\sum_{i=1}^{n}Oi}$$

where Pi is the simulated values, Oi is the observed values, n is the number of samples, and Ō is the mean of the observed data. The perfect statistics yield of good performance of model is indicated by E_NS_ = 1; RMSE = 0; CD = 1; and CRM = 0 [51].

### Calibration and validation of the models

The calibration of both models was performed after simulation to adjust parameters that affect runoff and sediment prediction in the studied site. The calibration was performed by using the maize monocropping (monocrop) with conventional practice (plowing layer > 15 cm). The conservation practice of intercropping (intercrop-hedgerow) high-value chili with *Leucaena* hedgerow and minimum tillage (plowing layer < 10 cm) was used for the validation to check the performance of the calibrated models (Fig 1). These treatments were calibrated and validated under the selected nine rainfall events that covered the growing season of maize in the year 2010 and covered periods of high rainfall intensity in order to minimize the effect of erratic rainfall distribution in the studied hillside area. In this study, the calibrated values are consistent with the values reported in the literature [39, 52]. Parameters that sensitively affect the runoff and sediment yield were modified for the best fit value between the observed and predicted value that showed in Table 4.

**Table 4:** Some sensitive parameters for calibration of the WaNuLCAS and WEPP models.

Model	Default value	Modified value
**WaNuLCAS**		
USLE_ERainFac	1	0.13
E_EntrailmentCoeffBarePlot	0.002	0.02
Rain_IntensCoefVar	0.3	0.08
Rain_IntensMean	50	28
Rain_IntercDripRt	10	9
Rain_PondFlwRt	10	9
Rain_PondStoreCp	5	4
S_BDBDRefDecay	0.0001	0.001
S_RelSurfInfiltrInit (Zone)	4	6
S_SurfInfiltrPerKsatDef (Zone)	0.0825	0.09
Maximum Leaf Area Index	10	6
Crop cover efficiency factor	0.2	0.1
Standard moisture content	0.5	0.15
**WEPP**	** **	** **
*Soil module*		
• Interrill erodibility: Ki (kg.s.m^-4^)	model calculation	250,000
• Rill erodibility: Kr (s.m^-1^)		0.0085
• Critical shear strength (Pa)		3.3
• Effective hydraulic Conductivity: Ksat (mm.h^-1^)		80
*Management module*		
• Tillage: chisel plow and no-till with fluted colter		
*Slope*		
Landforms of study’s area		12%

**Fig 2:**
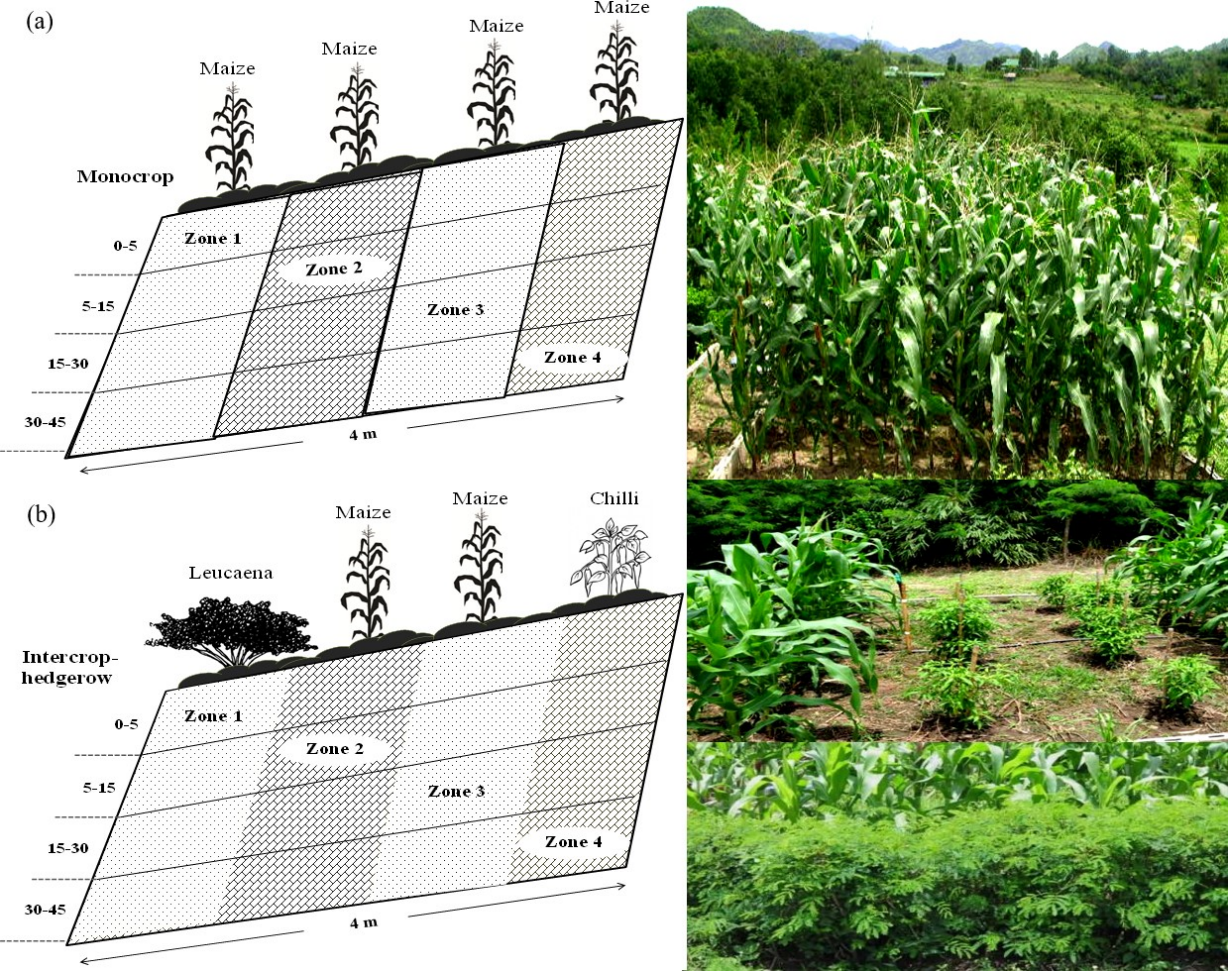
Scenarios of cropping systems for simulation of the WaNuLCAS and WEPP models.

The calibrations of the WaNuLCAS model was done after preparing input parameters for the site conditions. In the Excel (Microsoft Office) spreadsheets, the size and spacing of experimental plots were set up in four zones and layers of soil (Fig 2). Some soil inputs were filled in the pedotransfer sheet. Crop parameters in the crop library spreadsheet, crop, and management schedule in the crop management spreadsheet were modified. In the STELLA, soil dynamics was activated for soil structure change and some parameters in the soil structure module were modified. Parameters in the input sections of each module namely rainfall, sloping land, management, and soil erosion and sedimentation were also modified to achieve the best fit data.

For the WEPP model, the input parameters, interrill erodibility (K_i_), rill erodibility (Kr), critical shear stress (ρa), and effective hydraulic conductivity (K_sat_) were calibrated by adjusting to an acceptable value and also another section of the management file such as tillage and plant were selected from default crop parameter values for maize; this was extracted from the WEPP database [30] and some values were modified to fit data from the experiment. The coefficient of determination (R^2^) (1:1 linear correlation) was used for determining the sensitivity of the calibration and validation procedures.

## Results and discussion

### Effect of cropping systems and management practices on runoff and sediment yields

**Fig 3:**
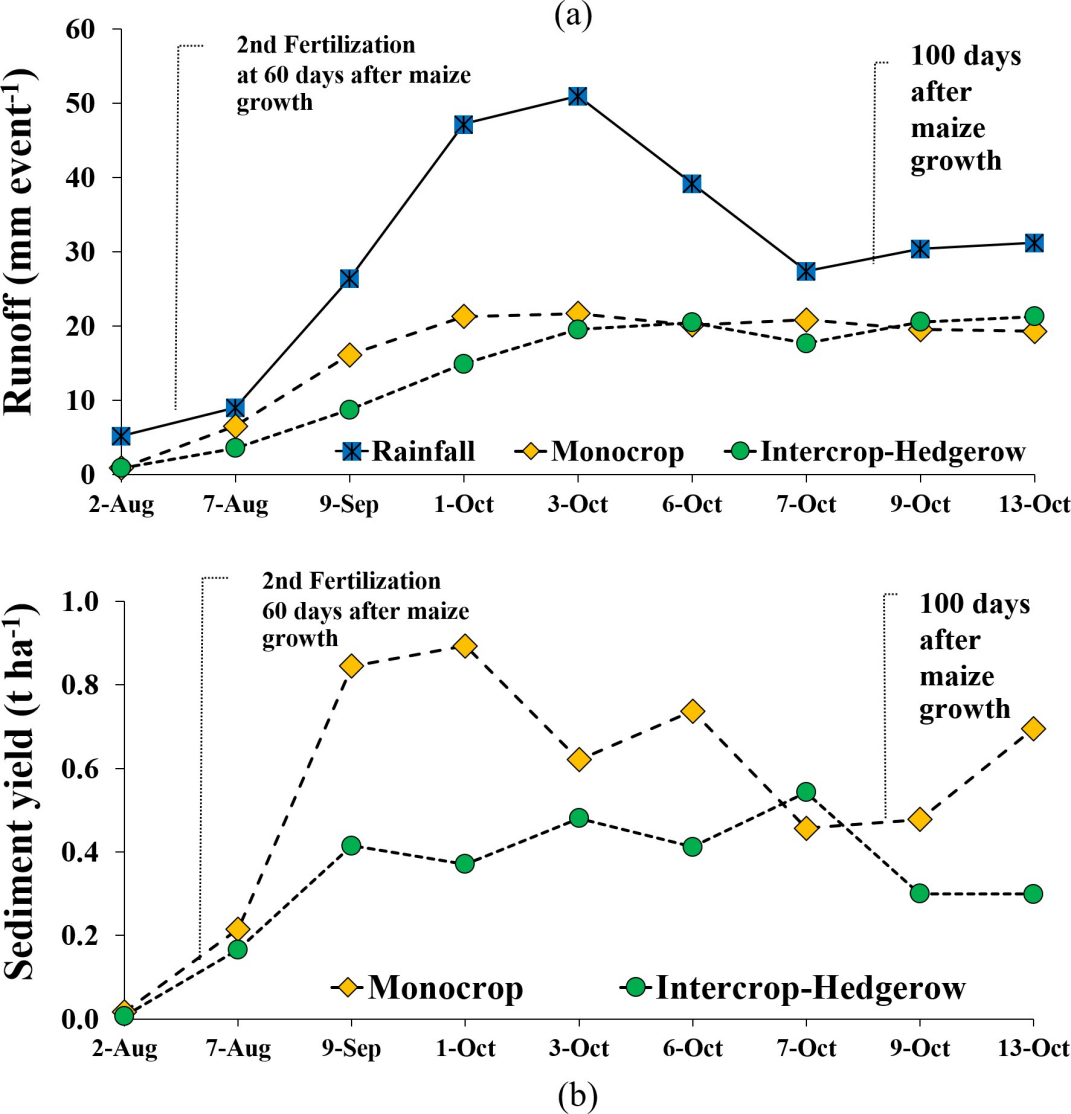
Runoff (**a**) and sediment yield (**b**) in monocrop and intercrop-hedgerow during the growing season of the year 2010.

Intercrop-hedgerow system could significantly reduce surface runoff and sediment as compared to monocropping with conventional practice. Intercrop-hedgerow system was capable of reducing surface runoff ranging of 15–45% and 23–60% as compared to the monocropping system during maize growing. Runoff yield (Fig 3A) (S1 Table) and sediment yield (Fig 3B) (S2 Table) and of the intercrop-hedgerow system were significantly lower than the monocrop system during August 7 to October 6, 2010, and corresponded to lower rainfall intensity with a high volume of rainfall. However, on the higher rainfall intensity periods (since October 7), the intercrop-hedgerow system had a slightly larger runoff volume and sediment yield than the monocrop system. This can be explained that on the aforesaid period, maize in monocrop system aged approximately 100 days was already ripe and ready for harvesting, which made the maize canopy density greater than that in the Intercrop-hedgerow system. Therefore, a higher canopy density of maize in the monocrop system increased rainfall-canopy interception in the monocrop during this period [53] and then reduced runoff and soil loss.

### The sensitivity of the WaNuLCAS model calibration

The sensitivity of the calibration parameters in the WaNuLCAS model (Table 5) revealed that the soil structure module and rainfall module were sensitive to runoff prediction. The most sensitive parameters for runoff prediction were enabling soil dynamics and erosion coefficient factors in the soil structure module. On the other hand, the most sensitive parameters affecting sediment yield prediction were crop parameters such as specific leaf area, plant canopy, root length density, rainfall interception, and crop cover efficiency factor. In addition, in this study, applying the USLE equation [54] to predict sediment yield was found to be more accurately predict soil loss than the ROSE equation did [55–57]. However, Wischmeier [58] warned against that using the USLE beyond the regions where the basic information was obtained, or to make soil loss estimates for individual erosion events. The USLE applies only to situations where net deposition does not occur. The USLE is based on correlations. As there is no inclusion in the USLE of factors directly representing physical parameters i.e., infiltration or overland flow velocity (ROSE equation), some factors will be influenced by correlations with effects caused by these processes. On the other hand, the modification of crop parameters in this study likely affected the infiltration rate and overland flow of rainwater. In addition, the variation of pore size distribution in this skeletal soil also directly affects the hydraulic conductivity and infiltration rate of soil which has been proved by Khetdan et al. [43].

**Table 5:** Sensitivity analysis for calibration parameters affecting runoff and sediment yield in the WaNuLCAS model.

Calibrated parameters in the WaNuLCAS	Runoff	Sediment yield
Default (soil dynamics enabled)	II	II
Rainfall module adjusted	I	I
Soil structure module (erosion coefficient factor)	II	I
Modified crop parameter	I	II

x, I, and II indicate non- sensitive, less sensitive, and more sensitive parameters, respectively.

As the sensitive parameters for runoff prediction were the soil structure module and rainfall module in the WaNuLCAS, soil structure refers to actual soil bulk density which calculated from the pedotransfer function from soil texture data (sand, silt, clay, and organic matter content) and involves directly with soil infiltration and saturated hydraulic conductivity of the soil. So, this study used soil bulk density derived from the pedotransfer function of the model which did not represent the actual values. Thus, the overestimation of runoff volume occurred by using the WaNuLCAS model. On the other hand, crop parameters were also the most sensitive parameters affecting sediment yield prediction by the WaNuLCAS model. Crop parameters also affect sediment yield and depend on the complicated of species and cultivars of the plant used in the system. So adjusting crop species parameters in the WaNuLCAS model could affect rainfall interception and crop canopy cover.

### The sensitivity of the WEPP model calibration

The sensitivities of the calibration parameters in the WEPP model are listed in Table 6; the most sensitive parameter in the WEPP model on runoff prediction was only the effective hydraulic conductivity (K_sat_). The parameters sensitive to sediment yield prediction were effective hydraulic conductivity (K_sat_), interrill erodibility (K_i_), and rill erodibility (K_r_); however, only K_i_ was the most sensitive parameter for sediment yield prediction. During the calibration process, the parameters in the soil file were calculated by the equation from USDA-ARS-MWA [30] using soil data from the experimental site and then modified until the output values closely fit observed data values.

**Table 6:** Sensitivity analysis for calibration parameters affecting runoff and sediment yield in the WEPP model.

Calibrated parameters in WEPP	Runoff	Sediment yield
Interrill erodibility (Ki)	x	II
Rill erodibility (Kr)	x	II
Critical shear strength (Pa)	x	x
Effective hydraulic conductivity (Ksat)	II	I

x, I and II indicate non- sensitive, less sensitive and more sensitive parameters, respectively.

The sensitivity analysis demonstrated that the interrill erosion was a dominant process occurred in the calibration system. So, the runoff and sediment prediction by the WEPP model depend directly on soil properties especially soil erodibility rather than crop parameters in the case of the WaNuLCAS model.

### Performances of the WaNuLCAS and WEPP models in runoff simulation

**Fig 4:**
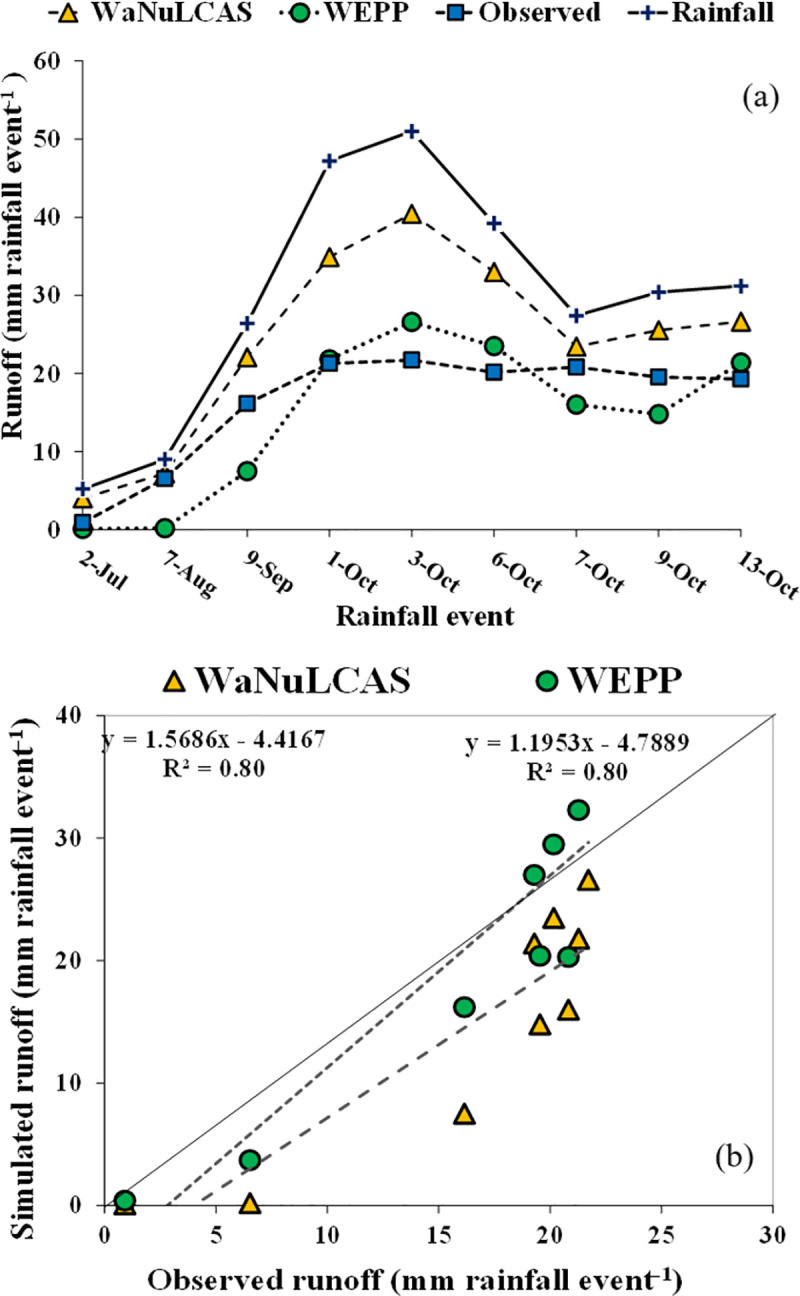
Model simulation for runoff prediction; (**a**) calibration of runoff in monocrop, (**b**)1:1 line relationship between simulated and observed runoff in monocrop.

**Fig 5:**
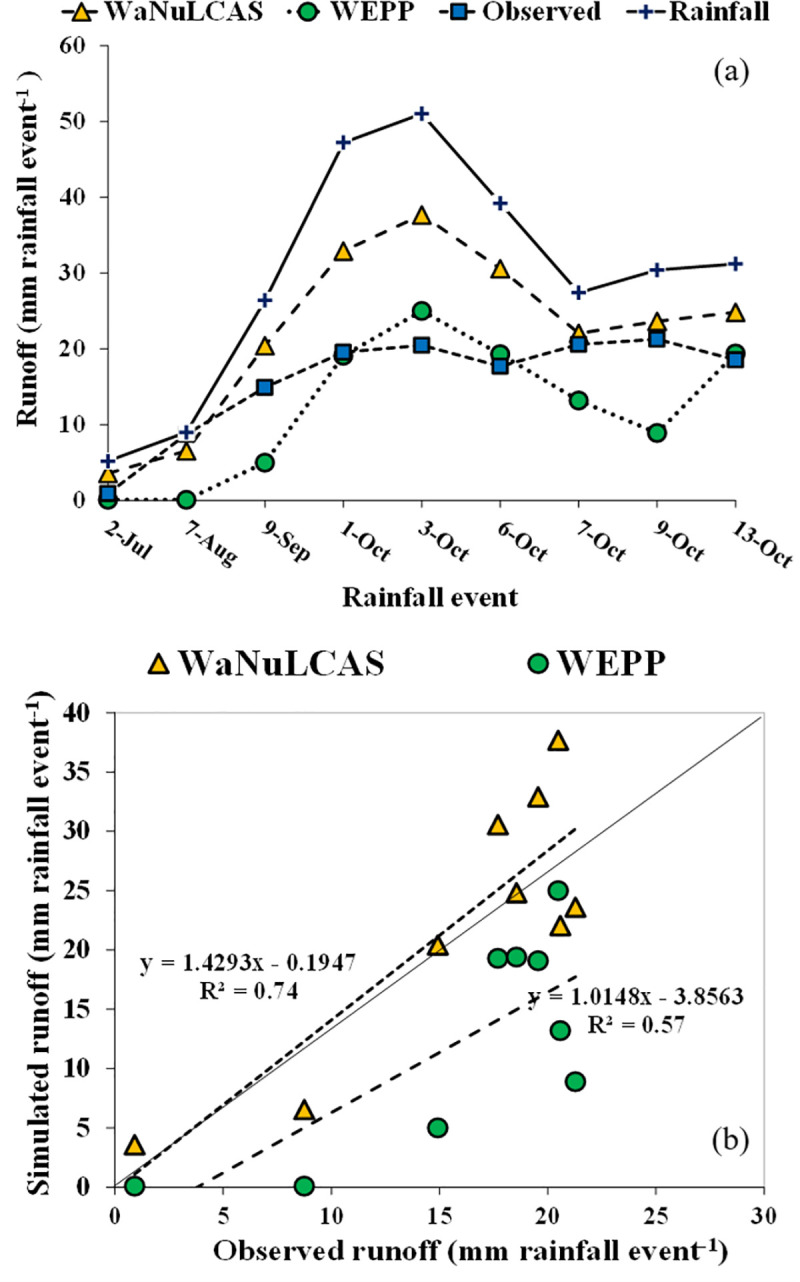
Model simulation for runoff prediction; (**a**) validation of runoff in intercrop-hedgerow (**b**) 1:1 line relationship between simulated and observed runoff in intercrop-hedgerow.

Simulation of daily runoff during the growing season by the WEPP and WaNuLCAS models was shown in Fig 4A for calibration (monocrop) and Fig 5A for validation (intercrop-hedgerow). The distribution of observed and simulated values along the 1:1 line for calibration and validation (intercrop-hedgerow) was shown in Figs 4B and 5B, respectively.

In Fig 4A, simulated runoff volume from the WEPP model was underestimated during the high rainfall, whereas during lower rainfall events the simulated runoff volume from the model was overestimated. All simulated runoff volume from the WaNuLCAS model was overestimated over all of the simulation period. The linear relationship between observed and simulated runoff values from both models was close to 1:1 line with R^2^ = 0.80 for the monocrop system (Fig 4B) and with R^2^ = 0.57 for the intercrop-Hedgerow system (Fig 5B). High R^2^ indicated that the WEPP model had a positive relationship between the observed and simulated runoffs. In addition, the RMSE and CRM expressed the error between the observed and simulated values, and the smaller values were found in the WEPP model indicating that the simulated values from the WEPP were closer to observed values than the simulated values from the WaNuLCAS model (Table 7). On the other hand, model efficiency in the runoff prediction was higher in the WEPP model (E_NS_ = 0.55), and it is more satisfactory for model performance as compared to the WaNuLCAS model (E_NS_ = –0.91). The negative model efficiency values indicated that the predicted values were greater than the observed values and the mean of the observed values [45]. This result confirmed that the WEPP model had a good performance for runoff prediction.

**Table 7:** The performances of the WaNuLCAS and WEPP model in runoff calibration and validation.

Models	Simulation	Mean runoff (mm)		Statistical analysis				
		Observed	Simulated	R^2^	E_NS_	RMSE	CRM	CD
**WEPP**	*Calibration*	146.41	131.90	0.80	0.55	4.73	0.10	0.56
	*Validation*	142.70	110.10	0.57	-0.09	0.36	0.23	0.47
**WaNuLCAS**	*Calibration*	146.41	217.23	0.80	-0.91	9.67	-0.48	0.26
	*Validation*	142.70	202.20	0.74	-0.73	9.00	-0.42	0.26

The underprediction of runoff by the WEPP model and over-prediction of runoff by the WaNuLCAS model were attributed to the different runoff calculation methods in the models. The WEPP model uses the GAML equation that considered excess rainfall in intervals of minutes and it is calculated as the difference between rainfall and infiltration rate. When rainfall intensity is smaller than the infiltration rate, water is stored in the soil [25]. In WaNuLCAS, runoff is primarily affected by the infiltration rate, which is obtained from the saturated hydraulic conductivity that uses soil physical properties calculated by pedotransfer functions on the van Genuchten equation [44]. The infiltration rate was affected by the saturated hydraulic conductivity that greatly impacts lateral and vertical water flows in the soil. Therefore, the WaNuLCAS considers the infiltration rate by the rainfall volume that affects soil saturated hydraulic conductivity, whereas the WEPP model considers infiltration rate by the intensity of rainfall. In addition, Pansak et al. [39] remarked that the WaNuLCAS model was capable of simulating the runoff and soil loss in Northeast Thailand reasonably well in maize-based upland cropping systems after careful calibration. Thus, the investigation of simulating the WaNuLCAS under the different climatic condition on the stony soil in this study needs more events for calibration and validation to verify the accuracy of data of the area under erratic rainfall pattern.

However, we attempt to validate both models in the intercrop-hedgerow system after model calibration to investigate the accuracy of our data. The results in Table 7 showed the R^2^, E_NS_, RMSE, CRM, and CD values of the runoff validation by the WEPP model were lower than the values from the calibration. While the R^2^, E_NS_, RMSE, CRM, and CD values in runoff validation by the WaNuLCAS model were close to the values in the calibration; however, both models can simulate a runoff volume in a similar trend in the monocrop system for overall rainfall events (Fig 4A). Nevertheless, many tested models have difficulties in simulating a low runoff. Chahinian et al. [59] attributed this difficulty to the problems inherent in determining the soil moisture conditions before and during flood events, which do not account for soil moisture redistribution over the whole duration of a flood event.

### Performances of the WaNuLCAS and WEPP models in sediment simulation

**Fig 6:**
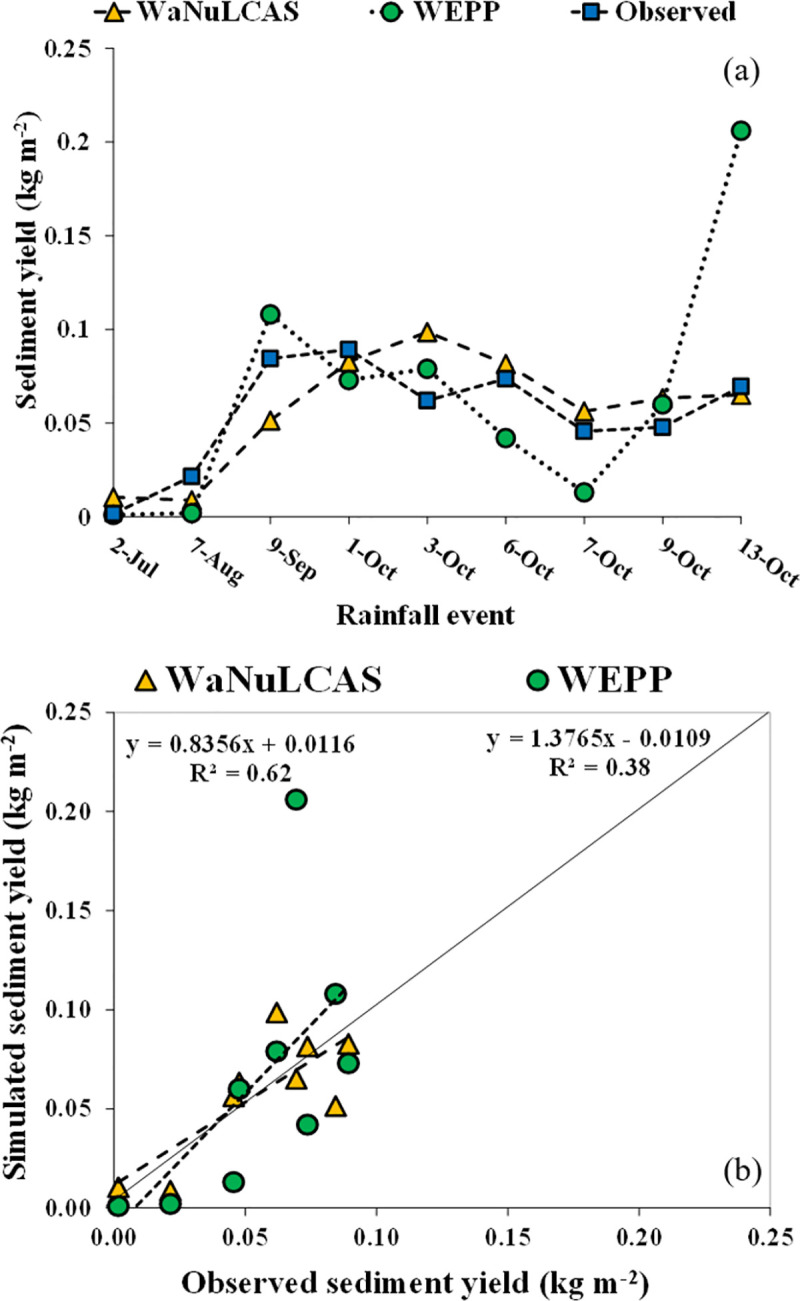
Model simulation for sediment yield; (**a**) calibration of sediment yield in monocrop, (**b**) 1:1 line relationship between simulated and observed sediment yield in monocrop.

**Fig 7:**
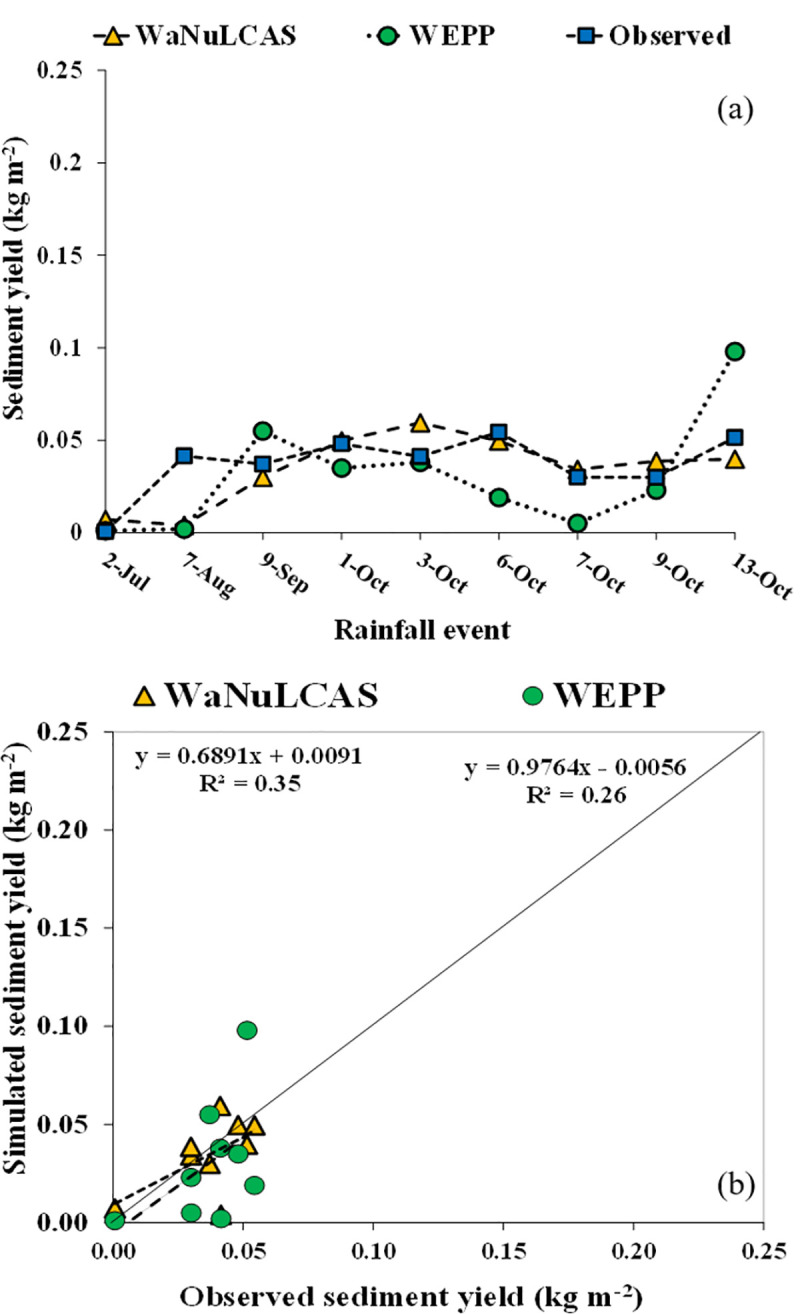
Model simulation for sediment yield; (**a**) validation of sediment yield in intercrop-hedgerow, (**b**) 1:1 line relationship between simulated and observed sediment yield in intercrop-hedgerow.

Simulation of daily sediment yield during the growing season in monocrop system by the WEPP and WaNuLCAS were shown in Fig 6A for the calibration (monocrop) and Fig 7A and for the validation (intercrop-hedgerow). The distribution of observed and simulated values along the 1:1 line for calibration (monocrop) and validation (intercrop-hedgerow) was shown in Figs 6B and 7B, respectively.

In Fig 6A, simulated sediment yield in monocrop system by WaNuLCAS model consistently matched with observed data except for a period of high rainfall volume (e.g., October 3, 2010) where the sediment yield was over-predicted to observed data, whereas the WEPP model simulated similar sediment yield to observed values along with the intensity of rainfall events which had a good performance similar to a prediction by the WEPP applied in the watershed scale in different climate condition [38]. In addition, the simulated sediment yield was distributed close to the 1:1 line (Fig 6B) indicating that both models had a positive relationship between the observed and simulated sediment yield. However, in the last rainfall event (October 3, 2010), the WEPP model showed the highest sediment yield. This was because maize was already ripe and had a low canopy and leaf area, and therefore, the highest soil loss occurred in this period. The simulation of sediment yield in intercrop-hedgerow system (Fig 7A) showed that the sediment yield predicted by the WaNuLCAS were closed to the observed value along with the rainfall events; however, the sediment yield predicted by the WEPP model was lower than the observed data in the periods between October 1 to 9. In addition, the simulated yield data were mostly above the 1:1 line which indicates that the simulated sediment yield was mostly higher than the observed sediment yield (Fig 7B).

The performance of the model simulation showed that the WaNuLCAS model precisely predicted sediment yield compared to the WEPP model, which indicates the model efficiency of sediment yield in calibration. The WaNuLCAS model predicted sediment yield with E_NS_ = 0.54, which is better than the model efficiency of the WEPP model (E_NS_ = –2.30), and the value of model efficiency is satisfactory for sediment yield calibration. Similarly, in the validation, the WaNuLCAS model could predict sediment yield better than the WEPP model indicating higher statistical values, as shown in Table 8. The WEPP model could perform better in the validation as compared to the calibration, whereas the WaNuLCAS model had lower performances in the validation. The results indicated that both models can predict sediment yield in the monocrop system better than that in intercrop-hedgerow. The difference in sediment prediction was caused by the different methods used in models where the WEPP model used the steady-sediment continuity equation to predict soil loss, whereas the WaNuLCAS model in this study used the USLE equation.

**Table 8:** The performances of the WaNuLCAS and WEPP models in sediment yield calibration and validation.

Models	Periods	Mean sediment yield (kg m^-2^)		Statistical analysis				
		Observed	Simulated	R^2^	E_NS_	RMSE	CRM	CD
**WEPP**	*Calibration*	4.96	5.84	0.38	-2.30	0.05	-0.18	0.20
	*Validation*	3.34	2.76	0.26	-1.89	0.03	0.17	0.27
**WaNuLCAS**	*Calibration*	4.96	5.18	0.62	0.54	0.02	-0.05	0.88
	*Validation*	3.34	3.12	0.35	0.10	0.02	0.06	0.80

The results of the performance assessment of both models confirmed that the WEPP model had a limitation in soil loss prediction in the intercropping system; however, it had a good performance in sediment prediction in the plot-scale monocropping system, and even in the watershed scale presented by Akbari et al. [38]. In contrast, the WaNuLCAS model had a good performance in soil loss prediction in the intercropping system. This was caused by the differences in plant parameters (i.e., leaf area, canopy, and plant species) and soil loss equation applied in the models [28, 29, 49].

## Conclusions

The WaNuLCAS and WEPP models were simulated to predict runoff and sediment yield on stony soil under selected tropical rainfall events in two different cropping systems: (i) maize monocropping with conventional practice and (ii) intercrop-hedgerow with conservation tillage, on hillside area in Suan Phueng district, Ratchaburi Province, Thailand. The most sensitive parameters for runoff simulation in the WaNuLCAS model were enabling soil dynamics and adjusting the parameter in the soil structure module, e.g., change in the soil layers. The most sensitive parameters affecting sediment yield in the WaNuLCAS model were crop parameters. For the WEPP model, the most sensitive parameter for runoff simulation was an effective hydraulic conductivity (Ksat), and for sediment yield simulation, it was effective hydraulic conductivity (Ksat), and interrill erodibility (Ki). Our results demonstrated that these parameters are the key site factors affecting performances of predicting soil loss and runoff by the WEPP and WaNuLCAS models in the tropical hillslope.

Based on statistical values of E_NS,_ RMSE, CRM, and CD, the WEPP model performed a satisfactory performance that was better than the WaNuLCAS model for runoff simulation. The WaNuLCAS model showed better performance of the sediment prediction than the WEPP model in calibration and validation. Thus, the WEPP model was more suitable for runoff prediction than sediment prediction, whereas the WaNuLCAS model was better for sediment yield prediction than runoff prediction in a tropical hillslope. However, our results imply that soil erosion prediction by these models needs a modification of default parameters in the modules especially soil and crop modules, and a collection of plants and crop parameters in each local scenario to estimate the accuracy of the models.

## Supporting information

S1 Table Simulated runoff values from the calibration and validation processes. (DOCX)

S2 Table Predicted sediment values from the calibration and validation processes. (DOCX)
