# Supplementary material for: Performances of the WEPP and WaNuLCAS models on soil erosion simulation in a tropical hillslope, Thailand
Source: PLoS One. 2020 Nov 4;15(11):e0241689. doi: 10.1371/journal.pone.0241689 (PMC7641452; doi:10.1371/journal.pone.0241689)
Supplement: S1 Table — (DOCX) [file pone.0241689.s001.docx]

**S1 Table.** Simulated runoff values from the calibration and validation processes.

| Rainfall event | Rainfall  from  datalogger (mm) | Observed runoff  yield  (mm) | Simulated runoff from  the calibration  (Maize-Monocrop)  (mm per rainfall event) | | Simulated runoff from  from validation  (Intercrop-hedgerow)  (mm per rainfall event) | |
| --- | --- | --- | --- | --- | --- | --- |
|  |  |  | WEPP | WaNuLCAS | WEPP | WaNuLCAS |
| 2-Jul-10 | 5.2 | 0.9 | 0.1 | 4.02 | 0.1 | 3.58 |
| 7-Aug-10 | 9.0 | 6.5 | 0.2 | 7.21 | 0.1 | 6.55 |
| 9-Sep-10 | 26.4 | 16.2 | 7.5 | 22.07 | 5.0 | 20.42 |
| 1-Oct-10 | 47.2 | 21.3 | 21.8 | 34.89 | 19.1 | 32.91 |
| 3-Oct-10 | 51.0 | 21.7 | 26.6 | 40.44 | 25.0 | 37.67 |
| 6-Oct-10 | 39.2 | 20.2 | 23.5 | 32.99 | 19.3 | 30.57 |
| 7-Oct-10 | 27.4 | 20.8 | 16.0 | 23.45 | 13.2 | 22.06 |
| 9-Oct-10 | 30.4 | 19.5 | 14.8 | 25.53 | 8.9 | 23.62 |
| 13-Oct-10 | 31.2 | 19.3 | 21.4 | 26.63 | 19.4 | 24.83 |
