# Supplementary material for: Performances of the WEPP and WaNuLCAS models on soil erosion simulation in a tropical hillslope, Thailand
Source: PLoS One. 2020 Nov 4;15(11):e0241689. doi: 10.1371/journal.pone.0241689 (PMC7641452; doi:10.1371/journal.pone.0241689)
Supplement: S2 Table — (DOCX) [file pone.0241689.s002.docx]

**S2 Table.** Predicted sediment values from the calibration and validation processes

| Rainfall event | Rainfall  from  datalogger (mm) | Observed  sediment yield  (ton ha^-1^) | Simulated sediment from  the calibration  (Maize-Monocrop)  (ton ha^-1^) | | Simulated sediment from the validation  (Intercrop-hedgerow)  (ton ha^-1^) | |
| --- | --- | --- | --- | --- | --- | --- |
|  |  |  | WEPP | WaNuLCAS | WEPP | WaNuLCAS |
| 2-Jul-10 | 5.2 | 0.001 | 0.001 | 0.010 | 0.001 | 0.007 |
| 7-Aug-10 | 9.0 | 0.041 | 0.002 | 0.009 | 0.002 | 0.004 |
| 9-Sep-10 | 26.4 | 0.037 | 0.108 | 0.051 | 0.055 | 0.030 |
| 1-Oct-10 | 47.2 | 0.048 | 0.073 | 0.083 | 0.035 | 0.050 |
| 3-Oct-10 | 51.0 | 0.041 | 0.079 | 0.099 | 0.038 | 0.059 |
| 6-Oct-10 | 39.2 | 0.054 | 0.042 | 0.082 | 0.019 | 0.050 |
| 7-Oct-10 | 27.4 | 0.030 | 0.013 | 0.056 | 0.005 | 0.034 |
| 9-Oct-10 | 30.4 | 0.030 | 0.060 | 0.063 | 0.023 | 0.039 |
| 13-Oct-10 | 31.2 | 0.051 | 0.206 | 0.065 | 0.098 | 0.040 |
